# Clinical efficacy of ureteroscopy-assisted laparoscopic ureteroplasty in the treatment of ureteral stricture after pelvic surgery

**DOI:** 10.1007/s11255-024-04115-4

**Published:** 2024-06-13

**Authors:** Jiansheng Xiao, Tairong Liu, Qiuhua Zhu, Liling Qiu, Jiaqi Ge, Hua Chen

**Affiliations:** 1 Department of Urology, Ganzhou People’s Hospital, Jiangxi Medical College, Nanchang University, Ganzhou, Jiangxi China; 2 Department of Operating Room, Ganzhou People’s Hospital, Jiangxi Medical College, Nanchang University, Ganzhou, Jiangxi China

**Keywords:** Laparoscopy, Ureteroscopy, Surgery, Ureteral stenosis, Pelvic surgery

## Abstract

**Objective:**

This study is to investigate the safety and efficacy of ureteroscope-assisted laparoscopic ureteroplasty in treating ureteral stricture after pelvic surgery.

**Methods:**

A retrospective analysis of the clinical data of 95 patients treated for ureteral stricture at Ganzhou People's Hospital from June 2017 to March 2023 after pelvic surgery. In this group, 49 patients underwent ureteroscope and laparoscopic ureteroplasty under lithotomy position. The control group consisted of 46 patients who underwent simple laparoscopic ureteroplasty in a supine position. Postoperative data from both groups were collected and compared, including operation time, amount of blood loss during surgery, postoperative hospital stay, incidence of complications, success rate of ureteroplasty, and effectiveness of the operation.

**Results:**

The success rate of end-to-end ureteral anastomosis in the observation group was 93.88%, and the operation effectiveness rate was 100%. The success rate in the control group was 78.26% and the operation effectiveness rate was 89.1%.The average operation time and intraoperative blood loss in the observation group were (121.3 ± 44.6) min and (137.5 ± 34.2) ml, respectively, while in the control group they were (151.2 ± 52.3) min and (165.6 ± 45.8) ml, the difference were statistically significant (*P* < 0.05). The incidence of perioperative complications in the observation group was 2%, significantly lower than that in the control group (19.6%) (*P* < 0.05).

**Conclusion:**

Ureteroscope-assisted laparoscopic ureteroplasty for ureteral stricture after pelvic surgery has the advantages of shortened operation time, increased success rate, and reduced incidence of complications, making it an optional surgical scheme in clinical practice.

## Introduction

With the rising incidence of tumoral diseases in the pelvis, the frequency of pelvic surgical interventions is also increasing. Consequently, postoperative ureteral stenosis following pelvic surgery has become more prevalent in clinical practice [1]. In addition, as the proportion of minimally invasive surgical interventions in urology is constantly increasing, holmium laser and other high-energy lithotripsy tools have become routine surgical instruments in urology, and the application of such lithotripsy tools in the treatment of ureteral incarcerated stones may also cause irreversible damage to the ureteral mucosa. Ureteral stenosis that occurs as a complication of lithotripsy of lower ureteral stones is becoming more common in clinical practice, and this type of ureteral stenosis is a challenge for physicians [2]. End-to-end ureteral anastomosis after resection of the ureteral stenosis segment is considered the most effective treatment for this type of stenosis [3]. However, due to previous surgical interventions, this type of surgeon often finds it difficult to locate the ureteral stenosis segment, the dissociation of the ureter in the pelvic area is also difficult because of surgical scars. Slightly careless injury to the normal ureter during the dissociation process may have serious adverse effects on the reconstruction of the patient's ureter [4]. To overcome these difficulties and improve the success rate of ureteroplasty, our hospital has adopted a combination of ureteroscopy and laparoscopic surgery in the lithotomy position in recent years, using ureteroscopy to assist laparoscopic ureteroplasty and obtained good clinical outcomes.

## Materials and methods

### Study population

In this study, we selected clinical data from 95 patients with ureteral stenosis after pelvic surgery from June 2017 to March 2023 in our hospital. Among them, 49 patients in the observation group underwent stone positioning ureteroscopy combined with laparoscopic ureteroplasty, 46 patients in the control group underwent simple laparoscopic ureteroplasty in the supine position. All patients underwent antegrade or retrograde urography, three-dimensional CT imaging of the urinary system, or ureteroscopic examination before surgery to confirm the presence of ureteral stenosis and fully clarify the location and length of the stenosis. The surgical approach is proposed by the chief surgeon based on an assessment of the ureteral stricture's location and length, considering the patient's surgical history and imaging results. The procedure is conducted after obtaining the patient's informed consent. Patients with ureteral stricture undergo segmental resection and end-to-end anastomosis. According to our center's clinical experience, for patients with a surgical history and a stricture segment longer than 3 cm, reanastomosis after segmental resection results in significant tension. Some patients even require the use of buccal mucosa, lingual mucosa, or appendiceal tissue as substitutes to restore ureteral continuity. Therefore, this retrospective study chose 3 cm as the threshold for long-segment ureteral stricture and used it as the inclusion and exclusion criteria for cases. Patients with a history of pelvic radiotherapy, multiple ureteral stenosis and long stenosis (stenosis length > 3 cm), stenosis section at the bladder wall or close to the wall estimated to be unable to undergo ureteral end-to-end anastomosis surgery, and severe hydronephrosis even pyonephrosis were not included in this study. This study was approved by the Ethics Committee of Ganzhou People’s Hospital, and informed consent was obtained from all patients, who signed informed consent forms.

### Surgical methods

**Fig. 1:**
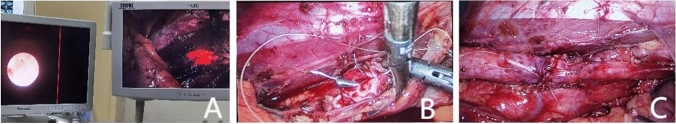
**A** Ureteroscope assisted laparoscopic localization of stenosis segment. **B** The posterior wall was sutured with the assistance of ureteroscope. **C** End to end ureter anastomosis was completed

All patients were put under general anesthesia. They were all placed Lithotomy position (head high and foot low). After routine pre-surgical disinfection and draping, the trocars were arranged according to the location of the patient's stenosis segment. A 10-mm Trocar was placed at the level of the umbilicus or the lateral border of the rectus abdominis muscle below the umbilicus as an observation hole. A 5-mm trocar and a 10-mm trocar were placed at the medial side of the anterior superior iliac spine, the intersection point between the umbilical level and the midclavicular line of the affected side, and about 0.5 cm from the affected side of the midpoint between the umbilicus and the affected anterior superior iliac spine. Initially, the paracolic sulcus was dissociated and the parietal peritoneum was opened; then, we found the normal renal pelvis or ureter above the ureteral stricture and moved down the normal renal pelvis and ureter to the scar adhesion. The adhesion is often serious and it is difficult to dissociate at this time. To minimize damage to the normal ureter, at this point, another assistant uses a ureteroscope under the patient to guide the ureteroscope under the zebra guidewire to the distal end of the ureteral stricture. The laparoscopic light source is turned off, the position of the ureteroscope light source is observed, the stricture segment is accurately located, and the narrow segment and normal ureter below the narrow segment are dissociated (Fig. 1A).

After fully dissociating the ureter, the ureteroscope was used again to support the position of the stricture. Using a pair of laparoscopic scissors close to the front of the ureteroscope, the distal end of the stenotic segment is cut first; then, the ureter toward the proximal end of the stenotic segment where ureteral dilatation is evident is slowly cut until urine is ejected. Then, after the normal mucosa of the ureteral lumen or normal mucosa of the renal pelvis could be seen, the proximal ureter was cut obliquely (If the lumen is small, cut 0.5–1 cm lengthwise at 8 o 'clock), and the distal normal ureter was cut 0.5–1.0 cm longitudinally at 3 o 'clock. With the assistance of ureteroscopy, the first three needles of the posterior ureteral wall were sutured with 4–0 absorbable suture (Fig. 1B), and then the zebra guidewire crossed the suture position into the renal pelvis. Under the monitoring of ureteroscopy and laparoscopy, the 7F double J tube was pushed retrograde. After withdrawing from the ureteroscope, the anterior ureteral wall was sutured with 4–0 absorbable sutures using 4–5 stitches intermittently. Appropriate reinforcement of the needle was performed to prevent urine leakage according to the suture situation (Fig. 1C). Complete hemostasis of the surgical area was performed after suturing was completed, and the drainage tube and urinary catheter were routinely placed beside the anastomosis.

The control group also used endotracheal intubation general anesthesia, and after catheterization, they were put in a supine position (head lower than feet) to perform simple laparoscopic ureteroplasty, 36 cases successfully carried out the removal of the narrowed section of the ureter and end-to-end anastomosis operation, Ten patients underwent ureter-bladder reimplantation, of which, three cases involved rolling the bladder muscle flaps, Normally, a drainage tube is placed next to the anastomosis.

### Postoperative management, follow-up, and efficacy evaluation

Patients should rest in bed for 1–3 d after surgery (depending on the anastomotic tension). Antibiotics should be used to prevent infection routinely, proper fluids should be administered to keep urine flowing well, prevent small blood clots, and prevent urine sediments from blocking the urinary catheter. Remove the abdominal drain when it is confirmed to be clean, remove the urinary catheter 2 weeks after it was placed, and return to the hospital three months later to remove the ureteral stent. Patients need to keep a urinary catheter in place for 2 weeks because early removal after complex ureteral stricture repair can lead to urine reflux and anastomotic leakage. Patients with bladder irritation symptoms may take M receptor blockers, such as tolterodine tartrate. Follow-up is conducted for 3 to 36 months after the tube is removed, with an average of (12.6 ± 5.3) months, patients return to the hospital one week after the double J stent is removed for a urinary system CT scan and kidney function test, patients return to the hospital 1 week after the double J stent is removed for a urinary system CT scan and kidney function test, if the treatment is effective, then a urinary system CT scan and kidney function test are performed every 3 months, with at least two consecutive checks.

### Observational indicators

1. Success rate and treatment effects of end-to-end ureteral anastomosis surgery performed on two groups of patients.
2. Perioperative indicators for both patient groups, including operation time, intraoperative blood loss, postoperative hospital stay, time of catheter removal, and occurrence of surgical complications.

### Evaluation of the therapeutic efficiency

All patients showed reduced renal hydronephrosis on CT of the urinary system 2 weeks after catheter removal. The following conditions are deemed effective: follow-up kidney function shows a decrease in creatinine levels compared to before; the patient's lumbar pain and other clinical symptoms have eased; renal hydronephrosis remains stable over two consecutive follow-ups.

### Statistical methods

Data were analyzed using SPSS 26.0 statistical software. Measurement data were expressed as mean ± standard deviation (*x* ± *s*) and subjected to a *t* test (when variances of two samples are equal) or an approximate *t* test (when variances of two samples are not equal). Count data were represented by the number of cases (%), and a *χ*^2^ test was performed. Differences with *P* < 0.05 were considered statistically significant.

## Results

### Population characteristics

There were no significant statistical differences in general clinical data such as gender, age, cause of stenosis, length of stenosis, and hydronephrosis between the observation group and control group, making them comparable (Table 1).

**Table 1:** Comparison of basic preoperative conditions between the observation group and the control group

Characteristics	Observation group (*n* = 49)	Control group (*n* = 46)	*t*/*χ*^2^	*P*
Gender (male/female)	15/34	16/30	0.187	0.664
Average age (years)	49.3 ± 8.6	47.4 ± 7.2	1.164	0.248
Narrow segment length (cm)	1.5 ± 0.8	1.4 ± 0.5	0.725	0.470
BMI^a^	24.2 ± 4.1	23.5 ± 4.3	0.812	0.419
Degree of renal pelvis dilatation (cm)	3.4 ± 1.3	3.2 ± 1.5	0.696	0.488
Position of narrow segment (left/right)	26/23	24/22	0.007	0.931

^a^Body mass index

### Comparison of completion rate, effectiveness, and incidence of complications of plastic surgery between patients in the observation group and the control group

In the observation group, 46 patients successfully underwent ureteroscopy combined with laparoscopic ureteroplasty, and 3 patients underwent ureteral-bladder reimplantation; in the control group, 36 patients successfully underwent laparoscopic ureteroplasty, and 10 patients underwent ureteral-bladder reimplantation, of which 3 patients underwent bladder muscle flap rolling, the success rate of ureteroplasty operation in the observation group, which was laparoscopic excision of narrowed ureter and end-to-end anastomosis, was higher than that in the control group (*P* < 0.05). In the observation group, only one patient developed significant gross hematuria after surgery, while in the control group, two patients developed urinoma, two patients developed postoperative fever, one patient developed vesicovaginal fistula, and one patient developed obvious gross hematuria; no patients in the observation group developed re-narrowing after catheter removal, with an effectiveness rate of 100%. The control group had three cases of re-narrowing and two cases of reflux symptoms, with an effectiveness rate of 89.13%. The total incidence of complications in the observation group was significantly lower than that in the control group, and the difference was statistically significant (*P* < 0.05) (Table 2).

**Table 2:** Comparison of completion rate, effectiveness, and incidence of complications of plastic surgery between patients in the observation group and the control group

Characteristics		Observation group (*n* = 49)	Control group (*n* = 46)	*t*/*χ*^2^	*P*
Completion rate of end to end ureter anastomosis (%)		46(93.8)	36(78.2)	4.898	0.026
Surgery effectiveness rate (%)		49(100)	41(89.1)	5.621	0.017
Postoperative complication rate (%)	Hematuria (cases)	1	1		
	Urinary incontinence (cases)	0	2		
	Fever (cases)	0	2		
	Vesicovaginal fistula (cases)	0	1		
	Re-stenosis (cases)	0	3		
	Total complication occurrence rate (%)	1(2.0)	9(19.6)	6.270	0.012

### Comparison of perioperative clinical data between observation group and control group patients

The average operation time and intraoperative blood loss in the observation group were less than those in the control group (*P* < 0.05). There was no significant statistical difference in the postoperative hospital stay and drainage tube removal time between the two groups (*P* > 0.05) (Table 3).

**Table 3:** Comparison of perioperative clinical data between observation group and control group patients

Characteristics	Observation group (*n* = 49)	Control group (*n* = 46)	*t*	*P*
Surgery duration (min)	121.3 ± 44.6	151.2 ± 52.3	3.004	0.003
Intraoperative blood loss (ml)	137.5 ± 34.2	165.6 ± 45.8	3.402	< 0.001
Postoperative hospital stay (d)	7.4 ± 1.2	7.8 ± 1.5	1.440	0.153
Time of drainage tube removal (d)	3.4 ± 1.5	3.6 ± 1.2	0.715	0.478

## Discussion

Currently, ureteral strictures are managed in a variety of ways [5]. Endovascular surgery is often the first choice for clinical physicians because of its minimally invasive nature and rapid associated postoperative recovery [6]. Many studies have shown that in patients with a history of pelvic surgery, endoluminal procedures (be they endotomy, balloon dilation, multiple stent retention, etc.) have poor long-term outcomes and may make patients undergo multiple operations [7–9]. Repeated ureteroscopic surgery will not only increase the difficulty of subsequent surgical procedures but also greatly increase the psychological and physiological burden of patients and also increase the economic burden on these patients [10]. Laparoscopic ureteral stricture resection and end-to-end anastomosis have been confirmed to be effective treatment options for such ureteral strictures that occur as a result of pelvic surgery [11]. However, after pelvic surgery, the ureteral stricture is often located and dissociated due to severe scar adhesion and normal anatomical stricture; thus, it can be difficult to free the ureter above and below the stricture. If the normal ureter is injured again during the process of dissociation, it may bring devastating consequences to the normal reconstruction of the ureter [12]. Therefore, accurately locating the ureteral stricture segment, safely dissociating the normal ureteral tissues above and below the stricture segment, and accurately cutting off the ureteral scar are critical to ensuring low-tension anastomosis of the ureter. They are also the difficult parts of the procedure [13].

In order to accurately determine the location of the ureteral stenosis, numerous scholars have tried different methods. Kim et al. [14] used indocyanine green near-infrared fluorescence imaging to accurately locate the narrow segment of the ureter; however, the indocyanine green dye may cause side damage to the ureter. Verbeek et al. [15] used low-dose methylene blue near-infrared fluorescence to guide the identification of ureteral stricture during surgery, however, due to the complexity of these methods, they have not been effectively promoted. There are also reports of scholars using flexible ureteroscopy to locate the ureteral stenosis during surgery, then using robot-assisted laparoscopy to treat lower ureteral stenosis, achieving good results [16–18].

In this study, we carried out laparoscopic ureteroplasty with the aid of a ureteroscope in the lithotomy position (head high and foot low), achieving good therapeutic results. We found during the surgery that freeing the dilated ureter above the stricture was relatively easier, but the ureter at and below the stricture often faced issues due to complications of previous surgeries, including intense inflammation, dense fibrotic scar tissue surrounding the ureter, and loss of normal anatomical structures, making the freeing process difficult [19, 20]. Therefore, after freeing the ureter above the narrow segment, we immediately let the assistant perform a retrograde ureteroscopic procedure from below. When the ureteroscope reached the narrowed segment through the affected ureter, we adjusted the brightness of the room and laparoscopic source to highlight the light from the ureteroscope, then used the guidance of the ureteroscope light and the assistance of the ureteroscope to free the normal ureter at and below the stenosis. The flexible use of ureteroscope can significantly reduce the difficulty of freeing during laparoscopic surgery, preventing further damage to the normal ureter and injury to surrounding organs, ensuring the safety of the surgery. Therefore, in this study, the average operation time and intraoperative bleeding volumes for the observation group were (121.3 ± 44.6) min and (137.5 ± 34.2) ml, respectively, compared to (151.2 ± 52.3) min and (165.6 ± 45.8) ml for the control group. The difference between the two groups is statistically significant.

When trimming the narrow segment, it should not be determined by the brightest location of the light source, but according to the position of the tip of the ureteroscope. Some patients, the narrow segment is not long, and the light can pass through it, where the brightest location is usually the normal ureter location. If the ureter is trimmed according to light intensity, it could inadvertently harm the normal ureter and affect the success rate and effectiveness of the surgery. Our method is to have the assistant press the tip of the ureteroscope against the narrow segment, touch the lens with the laparoscopic scissors, and trim the fibrous scar of the ureter along the front end of the ureteroscope lens, achieving precise trimming. After trimming the distal end of the narrow segment, the proximal end of the ureteral stricture is incrementally trimmed until the urinary flow is seen. During the ureteral suture, the ureteroscope can also play an auxiliary role. The ureteroscope passes through the trimming port and expands the ureteral cavity. When anastomosing the posterior wall of the ureter, the suture needle fastens throughout the ureter wall by following the ureteroscope, which can avoid accidentally encountering the anterior wall of the ureter, achieving precise anastomosis. The support from the ureteroscope is akin to an assistant inside the patient's abdominal cavity: the assistant can moderately swing or push the distal end of the ureter, helping the main surgeon achieve tension-free anastomosis. After the anastomosis of the posterior wall of the ureter is complete, the assistant uses the ureteroscope to retrogradely place a zebra guidewire to the renal pelvis, then withdraws the ureteroscope, and inserts a 7F double J tube retrogradely under the supervision of the laparoscope, ensuring that the ureteral stent is in the most rational position, playing an effective drainage role.

In this study, 49 patients in the observation group successfully underwent laparoscopic ureteral stricture segment resection and end-to-end anastomosis with the assistance of ureteroscopy. Among them, three patients underwent ureteral-bladder implantation due to an inability to perform end-to-end anastomosis because of the normal length of the ureter beneath the free ureteral narrowing segment after gynecological surgery. All surgeries were successfully completed. Only one patient had hematuria postoperatively, which recovered after conservative treatment, the complication rate was only 2%. Clinical symptoms improved, hydronephrosis decreased, and the effectiveness of the surgery reached 100% in the 49 patients followed up postoperatively. In the control group of 46 patients, 2 patients developed urinoma, 1 patient developed vesicovaginal fistula, and 2 patients developed postoperative fever. After the removal of the double J tube, three patients in the control group experienced re-narrowing. The postoperative complication rate was 19.6%, exhibiting a significant statistical difference when compared to the observation group. The surgical success rate and effectiveness rate in the observation group were significantly higher than those in the control group. Thus, it can be seen that ureteroscopy-assisted laparoscopic ureteroplasty can achieve efficient and safe surgical effects.

In this study, we summarized the following advantages of ureteroscopy combined with laparoscopy during ureteroplasty performed in the lithotomy position: first, the lithotomy position is convenient for the combined upper and lower operations required during surgery, and the surgeon and assistant each perform their duties to achieve mutual collaboration. Second, ureteroscopy can help the surgeon accurately dissociate the ureteral stricture segment, accurately locate the position of the ureteral scar, achieve fine tailoring of the stricture scar, and reduce the reduce side injury incurred during the operation. Third, ureteroscopy-assisted laparoscopic surgery can reduce the difficulty and tension of laparoscopic ureteral anastomosis, achieve the normal alignment of the ureteral mucosa and mucosa, avoid the formation of anastomotic scars, and prevent restenosis. Fourth, retrograde indwelling of the double J stent under laparoscopic direct vision and the indwelling zebra guidewire under ureteroscopy are capable of not only ensuring that the ureteral stent is placed in the best position but also saving the time spent on the laparoscopic placement of the double J stent and improving the efficiency of surgery.

However, this research has the following limitations: it is a single-center retrospective study with a relatively low number of surgical patients. Future prospective studies with more extensive data are expected to further confirm the safety and efficacy of this surgical approach.

## Conclusion

In conclusion, ureteroscopy-assisted laparoscopic ureteroplasty for ureteral stenosis after pelvic surgery is a safe and effective surgical method with certain clinical applications.

## Data Availability

The raw data supporting the conclusions of this article will be made available by the authors, without undue reservation.
